# Sulfonated Styrene‐Grafted Polyvinylidene Fluoride Copolymers for Proton Exchange Membranes for AQDS/Bromine Redox Flow Batteries

**DOI:** 10.1002/marc.202400852

**Published:** 2024-12-27

**Authors:** Francesca Niccolai, Elisa Guazzelli, Andrea Cesari, Zakaria El Koura, Ilaria Pucher, Giancarlo Galli, Elisa Martinelli

**Affiliations:** ^1^ Department of Chemistry and Industrial Chemistry University of Pisa Pisa 56124 Italy; ^2^ Green Energy Storage (GES) Povo Trento 38123 Italy

**Keywords:** fluorinated polymer, polyvinylidene fluoride, proton exchange membrane, redox flow battery, sulfonated polymer

## Abstract

This study presents the preparation and electrochemical testing of sulfonated styrene‐grafted poly(vinylidene fluoride) (pVDF) copolymers as proton exchange membranes (PEMs) for semi‐organic redox flow batteries (RFBs) based on 9,10‐anthraquinone‐2,7‐disulfonic acid (AQDS)/bromine. The copolymers are synthesized via a two‐step procedure, involving i) atom transfer radical polymerization of styrene (Sty) for the grafting to the pVDF backbone and ii) the sulfonation of the polystyrene grafted side chains. Copolymers with different amounts of sulfonated styrene (SSty) in the side chains (i.e., degree of sulfonation (*DS*)) are obtained and used for the preparation of PEMs by solution casting. The PEMs are characterized to assess their thermal, mechanical, water absorption, and ion exchange properties, to evaluate the effect of *DS* on membrane properties, and to select the membrane with the best overall performance for application in single cell tests. Electrochemical testing reveals that the pVDF‐*g*‐(Sty26‐*co*‐SSty14) membrane exhibits low crossover of redox species, favorable ohmic resistance, and energy efficiency. Results from single cell tests, as compared with commercial benchmarks such as Nafion 115 and Aquivion E87‐12s, highlight the potential of such copolymers as alternative membranes for RFBs.

## Graphical abstract

Sulfonated styrene‐grafted poly(vinylidene fluoride) proton exchange membranes, prepared by styrene grafting and subsequent sulfonation on a poly(vinylidene fluoride) backbone, demonstrate low crossover, favorable ohmic resistance, and high energy efficiency in a redox flow battery based on 9,10‐anthraquinone‐2,7‐disulfonic acid (AQDS)/bromine, outperforming commercial benchmarks like Nafion 115 and Aquivion E87‐12s, showcasing their potential as alternatives membranes for innovative energy storage systems.

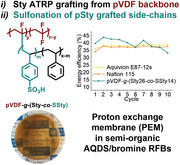

## 1 Introduction

In recent years there has been a great deal of interest in energy storage systems due to the growing demand for energy and increasingly looming environmental issues. Energy storage systems can be combined with intermittent renewable sources, such as solar and wind powers.^[^ ^1^, ^2^ ^]^ This provides a reduction in fossil fuels consumption and consequently in greenhouse gases emissions.^[^ ^3^ ^]^ In addition, energy storage systems ensure greater flexibility and stability of the distribution energy network and allow to save money by buying energy when it is cheaper. Furthermore, energy can be distributed even to locations that are isolated or far from the electrical grid.^[^ ^4^, ^5^ ^]^

Redox flow batteries (RFBs) have the feature of modulating separately energy capacity and power. Owing to this characteristic, they are considered attractive systems for energy storage.^[^ ^4^, ^6^, ^7^, ^8^ ^]^ Among the components of an RFB the polymeric membrane plays a crucial role as it determines performances and predominantly affects the total cost of the battery.^[^ ^9^, ^10^ ^]^ An ideal membrane should allow for high ionic conductivity, high selectivity, low ohmic resistance, limited crossover of redox‐active species, and good chemical and thermal resistance in aggressive environments. In addition, it should have low cost and be environmentally friendly.^[^ ^11^ ^]^

Membranes for RFBs are mainly divided into two categories, dense ion‐exchange membranes, and porous non‐ionic ones.^[^ ^12^, ^13^ ^]^ Membranes in the first category are characterized by the presence of charged groups that generally confer properties of high conductivity, high ion exchange capacity, and low crossover of active species, which are balanced by the high cost. According to the nature of the charged group, they are subdivided into cationic, anionic, or amphoteric.^[^ ^12^ ^]^ Non‐ionic porous membranes are generally characterized by higher mechanical stability and lower costs, but they suffer from low selectivity and high crossover of active species. Up to now, the most widely used membrane for redox flow batteries is Nafion, a proton exchange membrane (PEM), which is a perfluorinated random sulfonated polymer characterized by an electrically uncharged poly(tetrafluoroethylene) main chain with pendant perfluoropolyether side chains terminated by charged sulfonate end groups. The chemical incompatibility between the hydrophobic main chain and the hydrophilic side chains provides a phase separation, resulting in a well‐defined nano‐channel network.^[^ ^14^ ^]^ This morphology is generally accepted to be responsible for the highly effective proton transport across the membrane as well as its mechanical, thermal, and chemical resistance, associated also with the perfluorinated nature. However, the main disadvantage of Nafion membranes is their high cost, which prevents use for large‐scale energy storage.^[^ ^15^ ^]^

Therefore, there is a growing interest in developing novel, alternative, cost‐effective PEM membranes that satisfy the application requirements and possibly outperform the benchmark in terms of mechanical, chemical, and electrochemical properties. The development of new effective membranes requires choosing appropriate materials and optimizing synthetic procedures in order to obtain systems with well‐controlled morphology and structure. The type, number, and distribution of charged ionic groups as well as micro‐phase separation are mainly responsible for membrane characteristics such as ion exchange capacity, selectivity, permeability, water uptake, and chemical stability.^[^ ^16^, ^17^, ^18^ ^]^ Nowadays, the researchers' efforts are focusing on the use of alternative and highly efficient synthetic strategies, shifting the focus to (co)polymers and blends obtained via preparation methods that allow to combine the advantages of different materials in an additive or synergistic way, in order to prepare membranes that are equally or even better performing than the Nafion benchmark. Most of the literature on membranes for RFBs is based on the evaluation of membrane performance in vanadium RFBs (VRFBs)^[^ ^19^, ^20^, ^21^ ^]^ Although other RFB technologies may have different features, the membrane development principles for VRFBs are a source of inspiration for membranes used in other RFB configurations. Indeed, in any RFB technology, the membrane has mainly the role to ensure high ionic conductivity, and to minimize redox‐active species crossover, thus providing high selectivity and energy efficiency.^[^ ^19^, ^20^, ^21^, ^22^ ^]^

Commercially available fluorinated polymers, including poly(vinylidene fluoride) (PVDF), poly(vinylidene fluoride‐*co*‐chlorotrifluoroethylene) (PVDF‐CTFE), poly(vinylidene fluoride‐*co*‐hexafluoropropylene) (PVDF‐HFP) and their post‐functionalization derivatives are commonly reported as precursors for the preparation of ionic membranes for RFBs.^[^ ^23^, ^24^, ^25^, ^26^ ^]^ For example, PVDF‐based amphoteric membranes with styrene and dimethylaminoethyl methacrylate (DMAEMA) side chains were prepared by the grafting from approach, with gamma irradiation, for applications in VRFBs.^[^ ^23^ ^]^ Subsequent sulfonation of the styrene resulted in amphoteric membranes containing positive and negative charges. The final membrane composition was modulated by varying the ratio between the two monomers in the feed. The combination of the advantages of proton exchange membranes (higher conductivity) with those of anion exchange ones (higher selectivity) allowed to obtain membranes with a permeability of V(IV) of 1/10 with respect to that of Nafion and, at the same time, high conductivity. Sulfonated styrene and DMAEMA‐based side chains were also grafted onto ethylene tetrafluoroethylene copolymers (ETFE) for VRFB applications.^[^ ^23^ ^]^ Membranes derived therefrom showed lower permeability to vanadium ions and higher coulombic efficiency (*CE*) and overall energy efficiency (*EE*) with respect to Nafion 117, thus confirming their potential for applications in VRFBs.^[^ ^23^ ^]^ Membranes obtained by sulfonated styrene grafted to PVDF^[^ ^24^ ^]^ and PVDF‐HFP^[^ ^27^ ^]^ were also found to reduce the permeability of vanadium ions and improve the EE of the VRFB. Fluorine‐free specialty polymers are also attracting increasing interest because, in addition to their good chemical and thermal stability, they represent a solution to overcome the current concerns about the use of perfluorinated materials.^[^ ^28^ ^]^ Among the most widely investigated polymers for the preparation of membranes for RFBs are poly(ether sulfone) (PES),^[^ ^29^, ^30^ ^]^ poly(ether ketone) (PEK) or poly(ether ether ketone) (PEEK),^[^ ^31^, ^32^, ^33^ ^]^ polybenzimidazole (PBI)^[^ ^34^, ^35^ ^]^ and poly(phenylene oxide) (PPO).^[^ ^36^ ^]^ However, one of the major disadvantages of fluorine‐free membranes involves their reduced chemical and oxidative stability in harsh environment.^[^ ^37^ ^]^

Semi‐organic RFBs based on 9,10‐anthraquinone‐2,7‐disulfonic acid (AQDS)/bromine represent an innovative, promising, and emerging technology. The very few articles reported in literature are based on the commercial benchmark Nafion as ionic membrane only.^[^ ^38^, ^39^, ^40^, ^41^ ^]^ Therefore, the aim of the present work was to develop an alternative membrane to Nafion for possible use in AQDS/bromine RFBs. By taking into account the good properties and performance of sulfonated styrene grafted‐PVDF‐based membranes in fuel cells^[^ ^42^ ^]^ and FBs,^[^ ^24^ ^]^ we prepared and tested the first examples known of structurally related graft copolymers to devise PEMs for AQDS/bromine RFBs.

**Figure 1:**
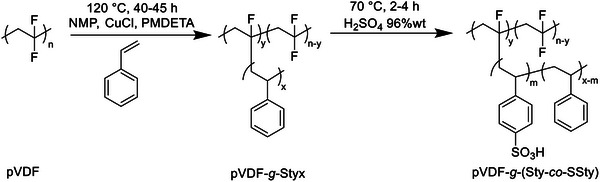
Synthesis of pVDF‐*g*‐Styx copolymers by ATRP (step 1) and sulfonation of graft copolymers (step 2).

The graft copolymers were prepared according to a two‐step procedure. In the first step, a commercially available polyvinylidene fluoride (pVDF) was post‐modified by grafting‐from polystyrene side‐chains via atom transfer radical polymerization (ATRP) (**Figure** **1**), while in the second step, the graft copolymers were subjected to a sulfonation reaction of the aromatic rings to incorporate the ‐SO_3_H group necessary to the proton transport (Figure 1). The sulfonated graft copolymers were then used for the preparation of PEMs, that were investigated from thermal, mechanical, and electrochemical viewpoints. Finally, the best‐performing membranes were subjected to AQDS/bromine single cell (4 cm^2^) test by performing a charge and discharge cycling study using the same electrolytes on both sides at different current densities.

## 2 Results and Discussions

### 2.1 Synthesis of the Graft Copolymers and Membrane Preparation

The sulfonated graft copolymers were prepared by a two‐step synthetic procedure (Figure 1). Specifically, in the first one, a commercially available pVDF was post‐modified by grafting‐from polystyrene side chains via atom transfer radical polymerization (ATRP). pVDF was selected as the backbone structure for its well‐known high chemical and thermal resistance as well as its excellent membrane forming capability. Moreover, the secondary fluorine of pVDF was found to be able to act as an initiating site for the ATRP of different monomers, including methacrylic acid, poly(oxyethylene methacrylate), sulfopropyl methacrylate and 4‐styrene sulfonic acid for the synthesis of amphiphilic graft copolymers as membrane precursors.^[^ ^43^, ^44^ ^]^ On the other hand, styrene (Sty) was our preferred low‐cost monomer offering the possibility of easy functionalization with sulfonic acid groups (–SO_3_H). The sulfonic acid is one of the most commonly used functional groups for PEMs able to effectively ensure ion‐exchange capacity, conductivity, and water uptake, thereby reducing the membrane resistance.^[^ ^7^, ^45^ ^]^

**Figure 2:**
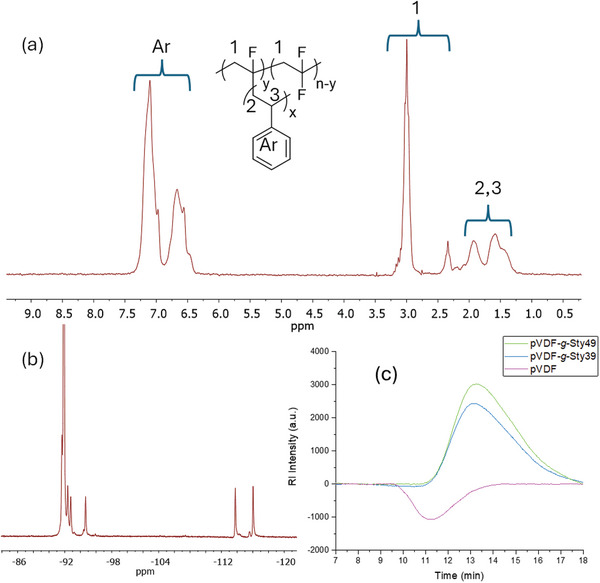
a) ^1^H‐NMR and b) ^19^F‐NMR spectra of copolymer pVDF‐*g*‐Sty49; GPC curves of pVDF and pVDF‐*g*‐Styx (*x* = 39 and 49 mol.%) in deuterated DMF obtained by c) RI detection.

The ATRP was carried out at 120 °C using copper chloride (CuCl) as the catalyst, *N*,*N*,*N*′,*N*′′,*N*′′‐ pentamethyldiethylenetriamine (PMDETA) as ligand and *N*‐methyl‐2‐pyrrolidone (NMP) as solvent. The success of the copolymerization was proved by both ^1^H‐NMR (**Figure** **2**a) and ^19^F‐NMR (Figure 2b) analyses (Figures S1 and S2, Supporting Information). The chemical composition of the final graft copolymer was estimated by taking into consideration the signals at 7.4−6.3 ppm of styrene aromatic units and the two regions at 3.3−2.8 and 2.5−2.35 ppm typical of pVDF main chain CH_2_ (Figure 2a; Figure S1, Supporting Information). The obtained copolymers are identified as pVDF‐*g‐*Styx where *x* is the molar percentage of Sty in the copolymer; this value was modulated in the range 29−51 mol.%. The properties of the prepared pVDF‐*g‐*Styx copolymers are collected in **Table** **1**. The number average molecular weight (*M*_n_) and dispersity (*Ð*) were evaluated by gel permeation chromatography (GPC) in DMF solutions. GPC measurements (Figure S3, Supporting Information) revealed the presence of a single, monomodal peak for graft copolymers pVDF‐*g‐*Styx, with an inversion in RI response and a different retention time from that of the pristine pVDF, suggesting that the styrene grafts were covalently anchored to the pVDF main chain (Figure 2c; Figure S3, Supporting Information). The incorporation of styrene side chains altered the polymer structure and affected its solubility in DMF with respect to pVDF, resulting in an apparent lower molecular weight of the graft copolymers (31 000 ≤ *M*_n_ ≤ 37 000 g mol^−1^, 1.96 ≤ *Ð* ≤ 2.24, RI detection) than that of the pristine pVDF (*M*_n_ = 169 000 g mol^−1^, *Ð* = 1.55) (Table 1). The values of dispersity were found to be generally high and not consistent with the expected controlled nature of the polymerization. This was possibly due to the high bonding energy of the C−F bond that led to a relatively slow initiation rate at this ATRP site to ensure an adequate control on the polymerization. The grafting of pSty side chains to the pVDF backbone was also confirmed by DOSY measurements, carried out on pVDF‐*g*‐Sty39 and pVDF‐*g*‐Sty49 graft copolymers, the pVDF starting material and two polystyrene samples with different molecular weights (19 000 and 233 000 g mol^−1^), taken as reference samples. In any case, one single diffusion coefficient (*D*) was evaluated (Table S1, Supporting Information), thus indicating that only one species was present in solution. In particular, *D* was 6.4 × 10^−11^ m^2^ s^−1^ and 2.8 × 10^−11^ m^2^ s^−1^ for pVDF‐*g*‐Sty39 and pVDF‐*g*‐Sty49, respectively. Such *D* values were similar when measured for peaks in the aromatic region, typical of pSty, and for CH_2_ protons, typical of the pVDF backbone, confirming the covalent grafting of pSty side chains. Moreover, *D* values of graft copolymers were lower than those of both pSty homopolymer samples and slightly higher than the one of pVDF starting material. This was in agreement with the GPC results that showed a lower molecular weight for copolymer with respect to pVDF in DMF. Finally, the solubility of the graft copolymers in both acetone (good solvent for pVDF and poor for pSty) and chloroform (good solvent for pSty and poor for pVDF) were found to be markedly improved with respect with those of the corresponding homopolymers. This supporting, although indirectly, the covalent anchorage of pSty chains to pVDF backbone.

**Table 1:** Chemical composition and properties of synthesized graft copolymers pVDF‐*g*‐Styx.

Graft copolymer	Sty^a)^ [mol.%]	Sty^a)^ [wt.%]	M_n_ ^b)^ [g mol^−1^]	Ð^b)^
pVDF‐g‐Sty51	51	71	31 000	2.04
pVDF‐g‐Sty49	49	68	31 000	2.08
pVDF‐g‐Sty39	39	53	36 000	1.96
pVDF‐g‐Sty29	29	40	37 000	2.24

^a)^ Molar and weight percentage of Sty counits in the graft copolymer evaluated by ^1^H‐NMR;

^b)^ Number average molecular weight and dispersity evaluated by GPC by using RI detection.

**Figure 3:**
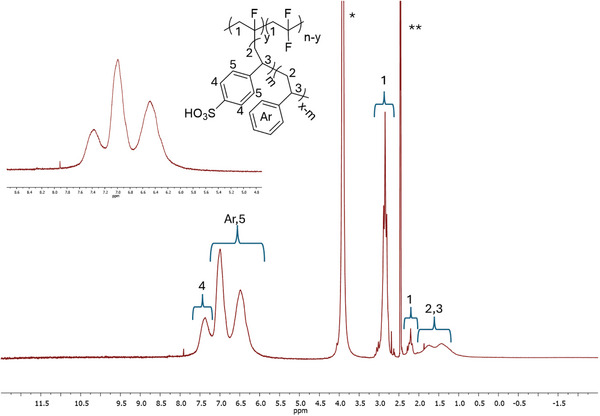
^1^H‐NMR spectra of pVDF‐*g*‐(Sty23‐*co*‐SSty14) copolymer in deuterated DMSO. Inset: signal at 6.0–7.8 ppm for Sty‐*co*‐SSty side‐chains.

**Figure 4:**
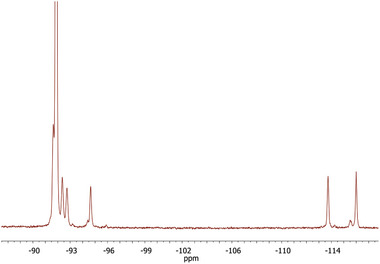
^19^F‐NMR spectra of pVDF‐*g*‐(Sty23‐*co*‐SSty14) copolymer in deuterated DMSO.

In the second step, the pVDF‐*g*‐Styx copolymers were subjected to a sulfonation reaction (Figure 1) in order to incorporate sulfonic acid functional groups (–SO_3_H) on the polystyrene grafts. The sulfonation reaction was carried out by dispersing the copolymer in 96 wt.% sulfuric acid at 70 °C, and the reaction time was varied to tune the mole percentage (*m*) of SSty counits in the graft copolymer and therefore the degree of sulfonation (*DS*) defined as the mole percentage of SSty in the Sty‐*co*‐SSty side chain grafts (**Table** **2**). *DS* was determined by ^1^H‐NMR (**Figure** **3**). ^19^F‐NMR spectra were also recorded of the post‐sulfonation graft copolymers (**Figure** **4**), but no significant difference was found between the spectra before and after sulfonation. Higher *DS* were obtained for a reaction time of 4 h for pVDF‐*g*‐Styx copolymers with similar initial content of styrene. Moreover, for a fixed sulfonation reaction time of 4 h, the polymeric precursor with a higher molar content of styrene achieved higher *DS*, e.g. a *DS* of 70% was reached starting from pVDF‐*g*‐Sty51, while *DS* was 53% from pVDF‐*g*‐Sty29. As is evident from Table 2, all the graft copolymers post‐sulfonation reaction were characterized by a total content of styrene comparable to the one before sulfonation. pVDF‐*g*‐(Sty6‐*co*‐SSty14) was the only sample with a marked lower content of styrene‐based counits significantly than the respective pVDF‐*g*‐Styx precursor. This is indicative of a greater fractionation of the crude product during the purification step by repeated precipitations in DI water, possibly due to the increased solubility of the copolymer in the DI water as a result of the sulfonation of a larger portion of styrene counits.

**Table 2:** Chemical composition and sulfonation degree (*DS*) of the sulfonated styrene graft copolymers.

Graft copolymer	Polymer precursor^a)^	Time [h]	pVDF^b)^ [mol.%]	Sty^b)^ [mol.%]	SSty^b)^ [mol.%]	DS^c)^ [%]
pVDF‐g‐(Sty26‐co‐SSty14)	pVDF‐*g*‐Sty49	2	60	26	14	35
pVDF‐g‐(Sty23‐co‐SSty14)	pVDF‐*g*‐Sty39	2	63	23	14	38
pVDF‐g‐(Sty6‐co‐SSty14)	pVDF‐*g*‐Sty51	4	80	6	14	70
pVDF‐g‐(Sty14‐co‐SSty16)	pVDF‐*g*‐Sty29	4	70	14	16	53

^a)^ Graft copolymer used as substrate for the sulfonation reaction;

^b)^ Mole percentage in the copolymer calculated by ^1^H‐NMR;

^c)^ Degree of sulfonation, that corresponds to the mole percentage of sulfonated styrene in the grafted chains.

The sulfonated styrene graft copolymers obtained were used for the preparation of films for PEMs by casting DMF solutions (12.5% w/v). The obtained films appeared free from macroscopic defects and homogeneous in thickness, that was in the range of 70−80 µm (Figure S4, Supporting Information).

### 2.2 Thermogravimetric Analysis

The thermal stability of the graft copolymers before and after the sulfonation reaction was analyzed by thermal gravimetric analysis (TGA). The TGA data are collected in Table S2 (Supporting Information).

**Figure 5:**
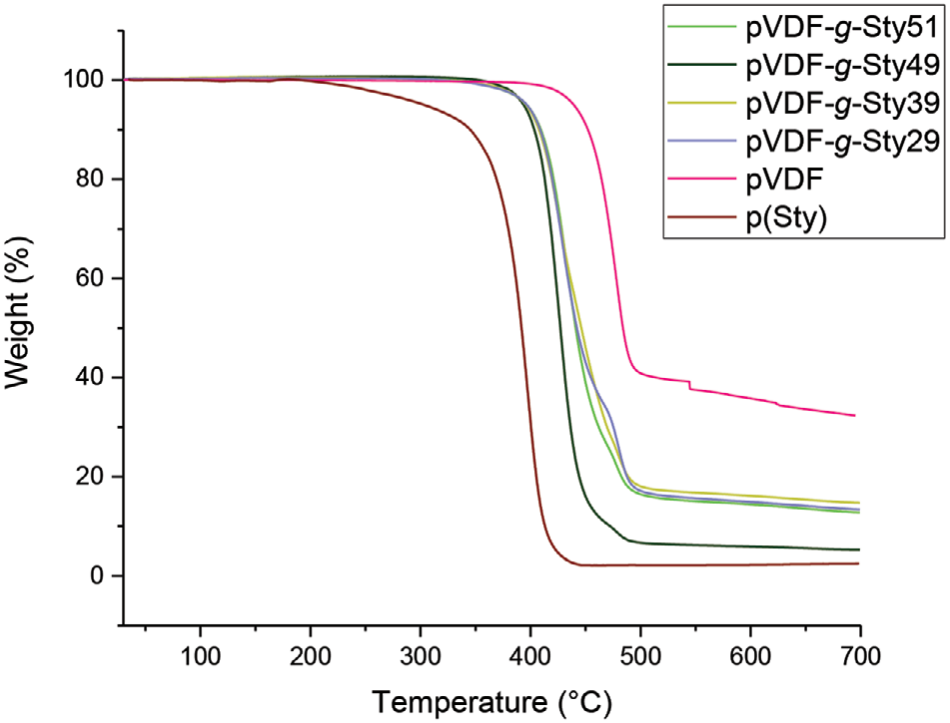
TGA curves of pVDF, pSty, and pVDF‐g‐Styx graft copolymers.

For pVDF‐*g*‐Styx copolymers, the TGA curves were characterized by a two‐step degradation (**Figure** **5**), with a temperature of initial degradation (*T*_onset1_) being lower than that of the pVDF homopolymer, thus indicating a reduced thermal stability of the graft copolymers with respect to the starting fluoropolymer. The first degradation step (*T*_max1_ = 422−431 °C) was mainly attributed to the degradation of the styrene‐based grafted chains, while the second step (*T*_max2_ = 465−480 °C) was mainly associated with the pVDF backbone, consistent with previous reports.^[^ ^46^ ^]^ This is also confirmed by the fact that the residue after the first step of degradation tended to decrease by increasing the content of styrene in the graft copolymer. As suggested by Hietala et al., this degradation pathway is in agreement with the view that the polystyrene side chains are incompatible with the pVDF matrix and form micro‐phase separated domains that behave distinctly upon degradation.^[^ ^46^, ^47^ ^]^ Moreover, the graft copolymers displayed a residue at 700 °C lower than that of pVDF.

**Figure 6:**
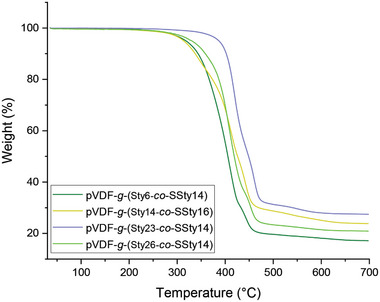
TGA curves of sulfonated styrene graft copolymers.

After the sulfonation reaction, the graft copolymers exhibited reduced thermal stability (*T*_max1_ = 408−420 °C) with respect to the respective non‐sulfonated counterparts (Table S3, Supporting Information; **Figure** **6**). However, their thermal stability was higher than that of structurally similar samples reported in literature that contained a higher mole percentage of sulfonated styrene counits.^[^ ^48^ ^]^ Desulfonation was proven to be the first step of degradation in this type of polymeric materials, occurring in the temperature range of 220−320 °C. However, for the graft copolymers reported here, the lower amount of sulfonated styrene counits as well as their copolymerization with the much thermally stable styrene appeared to prevent an earlier degradation of the copolymer. The two main degradation regions in the temperature ranges of 408−420 °C and 441−460 °C presented by the sulfonated graft copolymers were, therefore, associated with the degradation of the side and main chains, respectively.

The residue at 700 °C was higher for the sulfonated copolymers compared to the respective non‐sulfonated ones. Moreover, the residue showed an increasing trend with the sulfonation degree. This was consistent with what previously reported in literature and possibly due to an increase in char formation of the polystyrene grafts in the presence of water and sulfur dioxide (and other degradation products deriving from the sulfonic acid groups) formed during degradation.^[^ ^46^, ^49^, ^50^ ^]^

Overall, these results confirm that the membrane developed in this work were thermally stable at the operating conditions of an AQDS/bromine RFBs (*T* < 50 °C).

### 2.3 Differential Scanning Calorimetry Analysis

The thermal transition temperatures of the graft copolymers before and after sulfonation reaction were investigated by differential scanning calorimetry (DSC) and compared with those of the pVDF starting material. In particular, the latter was found to show a melting temperature (*T*_m_) at 170 °C and a glass transition temperature (*T*_g_) at −43 °C in agreement with the literature (**Table** **3**).^[^ ^51^ ^]^ The graft copolymers before sulfonation presented two glass transition temperatures and a melting temperature, thus preserving the semi‐crystalline nature of the pVDF starting material (Figure S5, Supporting Information). In particular, the low temperature *T*_g1_ (–52 °C to –47 °C) and the *T*_m_ (155–162 °C) were associated with the pVDF backbone, while the high temperature *T*_g2_ was attributed to the polystyrene side chains (Table 3). As expected, the degree of crystallinity (18–33%) was lower for the graft copolymers with respect to the pVDF homopolymer (42%) as a result of the incorporation of the grafted chains that interrupted the regular structure of the homopolymer. The results indicate that the pVDF‐*g*‐Styx graft copolymers were micro‐phase separated, because of the chemical incompatibility between the polystyrene and pVDF components.

**Table 3:** DSC data for pVDF and pVDF‐*g*‐Styx copolymers before and after sulfonation.

Polymer	DS [%]	T_g1_ ^a)^ [°C]	∆C_p_ ^b)^ [J g^−1^ K^−1^]	T_g2_ ^a)^ [°C]	∆Cp^b)^ [J g^−1^ K^−1^]	T_m_ ^a)^ [°C]	∆H/g^c)^ [J g^−1^]	Crystallinity^d)^ [χ%]
pVDF		−43	0.11			170	44.1	42
pVDF‐g‐Sty49		−52	0.09	92	0.22	158	13.3	33
pVDF‐g‐(Sty26‐co‐SSty14)	35	−44	0.07	111	0.10	168	13.5	31
pVDF‐g‐Sty39		−50	0.04	91	0.19	162	9.3	18
pVDF‐g‐(Sty23‐co‐SSty14)	38	−44	0.07	112	0.13	167	10.9	23
pVDF‐g‐Sty29		−47	0.07	86	0.24	155	12.8	20
pVDF‐g‐(Sty14‐co‐SSty16)	53	−44	0.08	114	0.11	170	15.1	29
pVDF‐g‐Sty51		−52	0.05	87	0.20	157	13.0	33
pVDF‐g‐(Sty6‐co‐SSty14)	70	−42	0.08	113	0.14	170	17.9	28

^a)^ *T_g_*_1_, *T*_g2_, and *T*_m_ values from the second heating cycle;

^b)^ Heat capacity variation corresponding to glass transition temperature;

^c)^ Experimental enthalpy of fusion;

^d)^ Degree of crystallinity calculated as the ratio between the measured enthalpy of fusion normalized with respect to pVDF wt.% content and the enthalpy of fusion of a 100% crystalline pVDF (104.5 J g^−1^).^[^ ^53^ ^]^

For the graft copolymers after sulfonation reaction, the same three thermal transitions were also observed, even though *T*_g2_ was generally higher (111–114 °C), because of the strong electrostatic interactions among the charged groups (Table 3, Figure S5, Supporting Information). Moreover, the melting temperature of the pVDF component tended to increase after sulfonation (e.g.*, T*_m_ = 157 °C and 170 °C for pVDF‐*g*‐Sty51 and pVDF‐*g*‐(Sty6‐*co*‐SSty14), respectively), suggesting that the incorporation of SSty counits enhanced the chemical incompatibility between the backbone and the grafted chains. In addition, the sulfonated graft copolymers presented a degree of crystallization comparable to or even higher than that of the corresponding non‐sulfonated counterparts. The increase in *T*_m_ in structurally similar pVDF‐*g*‐SSty copolymers was previously attributed to the strong interactions of ‐SO_3_H groups, which allows for the formation of micro‐phase separated domains.^[^ ^52^ ^]^

### 2.4 Mechanical Properties of the Membranes

**Figure 7:**
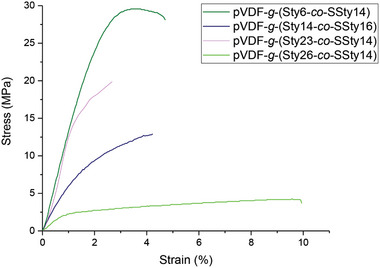
Stress–strain curves for sulfonated styrene graft copolymers.

Membranes should possess a suitable mechanical resistance to overcome the assembly stage of the cell and withstand the cell operating conditions while avoiding fracture or breakage. The mechanical properties of membranes were evaluated by performing tensile stress‐strain tests after conditioning the dog bone‐shaped specimens in DI water for 24 h (**Figure** **7**). Evaluation of the mechanical properties under wet conditions was preferred on account of the operating conditions of real cells. The values of Young's modulus (*E*), maximum stress (*σ_max_*), and elongation at break (*ε*) of the tested membranes are collected in Table S4 (Supporting Information). Although the content of SSty was similar for all the graft copolymers, the values of *E* were found to significantly increase by increasing the degree of sulfonation. For example, *E* rose from 220 MPa to 1085 MPa by increasing *DS* from 35% to 70%. On the other hand, *ε* was generally low and similar for all the samples. Moreover, the elongation of the samples up to fracture did not result in macroscopic necking. No membrane was found to break or crack after the cell test, suggesting that the mechanical properties were suitable to withstand the cell operating conditions.

### 2.5 Water Uptake, Ion Exchange Capacity, Ex Situ and In Situ Electrochemical Properties of the Membranes

Water uptake (*WU*) is a very important parameter to evaluate in ion exchange membranes as it impacts mechanical properties,^[^ ^54^, ^55^ ^]^ proton conductivity,^[^ ^56^ ^]^ permeability of the redox‐active species, and consequently on the overall performance of membranes in RFBs.^[^ ^57^ ^]^ The interaction of charged ionic groups with water influences the formation of water channels where proton transport occurs, so that proton mobility is affected by water content and structure.^[^ ^58^ ^]^ Increased *WU*, generally, promotes proton conductivity and permeability, but negatively affects mechanical properties as high *WU* values result in high swelling and plasticization of the polymeric membrane. Optimization of *WU* is therefore important to balance the described properties and improve performance in the final device. However *WU* in ion exchange membranes is strictly dependent on the ion exchange capacity (*IEC*), that is the number of ionic groups available, capable of exchanging ions, in the membrane. In particular, *WU* increases by increasing *IEC*. Moreover, also conductivity increases by increasing *IEC*. Therefore, in an ion exchange membrane, all these parameters should be balanced in order to obtain materials in which conductivity is maximized without significantly increasing redox‐active species permeability and reducing mechanical and dimensional stability.^[^ ^19^, ^20^, ^21^, ^22^ ^]^

**Figure 8:**
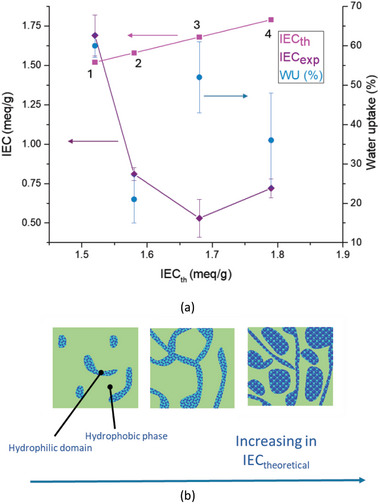
a) *IEC*_exp_, *IEC*_th_, and *WU* as functions of *IEC*_th_ for pVDF‐*g*‐(Sty26‐*co*‐SSty14) (1), pVDF‐*g*‐(Sty23‐*co*‐SSty14) (2), pVDF‐*g*‐(Sty6‐*co*‐SSty14) (3), and pVDF‐*g*‐(Sty14*‐co*‐SSty16) (4); b) schematic illustration of the micro‐phase separation in the PEMs as a function of the increase in *IEC*_th_.

The *WU* and ion exchange capacity (*IEC*_exp_) of the sulfonated membranes, along with those of two commercial benchmarks (Nafion 115 and Aquivion E87‐12s), were measured and collected in **Table** **4**. Generally, the sulfonated membranes had lower *IEC*_exp_ and higher *WU* compared to the benchmarks. **Figure** **8**a shows *IEC*_exp_ versus theoretical ion exchange capacity (*IEC*_th_), based on the mole amount of SSty counits in the copolymers.

**Table 4:** Water uptake (*WU*), theoretical (*IEC*_th_), and experimental (*IEC*_exp_) ion exchange capacity for graft copolymer‐based membranes and two commercial benchmarks.

*Membrane*	*WU* [%]	*IEC_th_* [meq g^−1^]	*IEC_exp_* [meq g^−1^]
*Nafion 115*	17 ± 2	0.85	0.84 ± 0.01
*Aquivion E87‐12s*	20 ± 1	1.05	1.06 ± 0.04
*pVDF‐g‐(Sty6‐co‐SSty14)*	52 ± 9	1.68	0.53 ± 0.12
*pVDF‐g‐(Sty14‐co‐SSty16)*	36 ± 12	1.79	0.72 ± 0.06
*pVDF‐g‐(Sty23‐co‐SSty14)*	21 ± 6	1.58	0.81 ± 0.04
*pVDF‐g‐(Sty26‐co‐SSty14)*	60 ± 3	1.52	1.69 ± 0.13

Notably, sulfonated styrene graft copolymers with higher degrees of sulfonation (*DS*) of 70% and 53% had much lower *IEC*_exp_ than expected. In contrast, the sample pVDF‐*g*‐(Sty26‐*co*‐SSty14) with a lower *DS* of 35% had *IEC*_exp_ and *IEC*_th_ that matched and were higher than the benchmarks. This suggests that *IEC*_exp_ does not increase consistently with *IEC*_th_. Above an *IEC*_th_ value of 1.52 meq g^−1^, further increases in DS and *IEC*_th_ did not boost *IEC*_exp_ but instead decreased it, reducing proton exchange efficiency. This is due to the aggregation of ionic sites into large clusters, which reduces the number of sites available for forming well‐connected hydrophilic channels necessary for effective ion transport (Figure 8b). *WU* values ranged from 21% to 60% and did not clearly correlate with *IEC*_exp_, although the membrane with the highest *IEC*_exp_ also had the highest *WU* (Figure 8a).

The transport of active redox species across the membrane is an issue that affects the overall performance of the cell, leading to a loss of capacity and energy efficiency. Therefore, crossover is a critical phenomenon that must be evaluated and reduced for the development of membranes for RFBs.^[^ ^59^ ^]^ Specifically, through potentiostatic measurements, the crossover of AQDS and HBr across the membrane pVDF‐*g*‐(Sty26‐*co*‐SSty14) with the highest *IEC*_exp_ was evaluated *ex situ* and compared with those of Nafion and Aquivion benchmarks (**Table** **5**). All membranes showed a lower crossover of AQDS compared to the brominated species, given the larger steric hindrance of the organic molecule. In addition, the pVDF‐*g*‐(Sty26‐*co*‐SSty14) membrane displayed the lowest crossover values for both HBr and AQDS species, thus representing an improvement with respect to the benchmarks whose major drawback is species crossover over time.

**Table 5:** Crossover of HBr and AQDS for Nafion, Aquivion and pVDF‐*g*‐(Sty26‐*co*‐SSty14) membranes.

Membrane	Crossover HBr [mol cm^−1^ s^−1^]	Crossover AQDS [mol cm^−1^ s^−1^]
Nafion 115	2.44 × 10*^−^* ^09^	6.3 × 10*^−^* ^11^
Aquivion E87‐12s	2.40 × 10*^−^* ^09^	1.5 × 10*^−^* ^11^
pVDF‐g‐(Sty26‐co‐SSty14)	1.37 × 10*^−^* ^10^	1.45 × 10*^−^* ^11^

Finally, a charge–discharge cycling study was conducted at different current density in order to ascertain the effective potential of the membrane prepared. The experiments were carried out in 1 W test cell, by using the same electrolytes (0.5 m AQDS, 1.5 m HBr, 0.165 m MEP), and electrode (carbon cloth) in both half cells, and by performing ten cycles at 50 mA cm^−2^ followed by ten cycles at 100 mA cm^−2^. Membrane performance was analyzed by evaluating energy efficiency (*EE*). The *EE* (calculated according to Equation (1)) provides information on the energy obtained during the discharge process versus that required in the charging process, thus providing a measure that overall describes the operation of the cell.

(1) $$EE=\frac{Discharge\;power\;\left(Wh\right)}{Charge\;power\;\left(Wh\right)}\;\cdot 100\;$$

**Figure 9:**
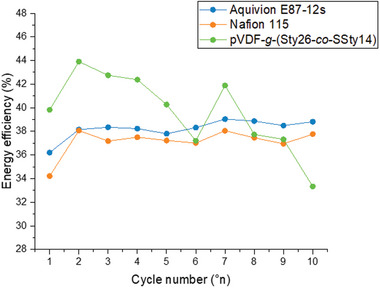
Energy efficiency (*EE* %) of pVDF‐*g*‐(Sty26‐*co*‐SSty14), Nafion 115 and Aquivion E87‐12s for ten cycles at 100 mA cm^−2^.

The cell resistance with pVDF‐*g*‐(Sty26‐*co*‐SSty14) was found to be 100 m𝛀, lower than that of both Nafion 115 and Aquivion E87 12 s (500 m𝛀). The *EE* of the pVDF‐*g*‐(Sty26‐*co*‐SSty14) membrane was equal to or greater than that of the benchmarks for the last ten cycles at 100 mA cm^−2^ (**Figure** **9**). All these properties make pVDF‐*g*‐(Sty26‐*co*‐SSty14) membrane a promising candidate for application in semi‐organic RFBs.

## 3 Conclusion

In conclusion, graft copolymers pVDF‐*g*‐Styx composed of a pVDF backbone and polystyrene side chains were effectively synthesized by ATRP. The Sty grafts were then successfully converted into Sty‐*co*‐SSty grafts containing sulfonated styrene counits randomly distributed in the side chains simply by treatment with concentrated sulfuric acid. The relative amount of SSty counits was tuned by changing the reaction time. DSC analysis revealed that the membranes were phase separated in bulk into pVDF domains and charged Sty‐*co*‐SSty domains, that were responsible for the proton exchange. However, the *IEC*_exp_ was found to be the highest for the sulfonated graft copolymers with the lowest *IEC*_th_, indicating that a relatively high content of SSty in the side chains did not improve proton exchange and transport. The pVDF‐*g*‐(Sty26‐*co*‐SSty14) with the highest *IEC*_exp_ was selected for cell tests. Interestingly, such a membrane was found to display a lower crossover of both AQDS and brominated species than those of two commercial benchmarks. Charge‐discharge cycling tests carried out with the same electrolytes at both sides of the cell revealed that the membrane exhibited favorable ohmic resistance, low‐capacity loss, and higher energy efficiency comparable with that of the benchmarks. These results indicate that PEMs based on the sulfonated graft copolymers developed in this work are promising for the AQDS/bromine RFB technology and warrant further investigations in the quest for potential alternatives to the commercially available membranes. To the best of our knowledge, this represents the first example of an alternative membrane to Nafion for application in semi‐organic AQDS/bromine RFBs.

## 4 Experimental Section

### Materials

Dimethylformamide (HPLC grade, Sigma‐Aldrich, Darmstadt, Germany), 96% sulfuric acid (Carlo‐Erba, Cornaredo (MI), Italy), methanol (Carlo Erba, Cornaredo (MI), Italy), 48% hydrobromic acid (Sigma‐Aldrich, Darmstadt, Germany), *N*‐ethyl‐*N*‐methyl pyrrolidinium bromide (MEP) (Sigma‐Aldrich, Darmstadt, Germany) and deuterated solvents (Sigma‐Aldrich, Darmstadt, Germany) were used without further purification. *N*‐methyl‐2‐pyrrolidone (NMP) (Sigma‐Aldrich, Darmstadt, Germany) was left overnight on molecular sieves, then distilled under vacuum and stored under N_2_. Copper chloride (CuCl) was suspended in acidic water (few drops of 37% HCl added to deionized water) to obtain a white solid that was repeatedly washed with water, then ethanol and diethyl ether. Finally, the product was dried under vacuum and stored under N_2_. Styrene (Sty) (98% purity) was purchased from Sigma Aldrich (Darmstadt, Germany) and purified via washings with 5% NaOH and water to remove inhibitors. After drying over Na_2_SO_4_, it was distilled under reduced pressure and stored under N_2_. *N*,*N*,*N*′,*N*′′,*N*′′‐pentamethyldiethylenetriamine (PMDETA) (Sigma Aldrich, Darmstadt, Germany) was distilled under vacuum and stored under N_2_. Poly(vinylidene fluoride) (pVDF) was purchased from Ambofluor (Ambofluor GmbH & Co. KG, Hamburg, Germany) with a declared number average molecular weight of 250−300 kg mol^−1^. 2,7­Anthraquinone disulfonic acid disodium salt (Riverside Specialty Chemicals, New York, USA) was dissolved in deionized water and repeatedly passed through amberlite H‐120 and then dried to obtain 2,7­anthraquinone disulfonic acid (AQDS). Nafion 115 was purchased from Ion Power (Munchen, Germany), while Aquivion E87‐12s was purchased from Solvay specialty polymers (Italy).

### Characterization

^1^H‐NMR, ^19^F‐NMR spectra were recorded with JEOL CZR 500 MHz and JEOL YH400 MHz spectrometers. Samples were prepared by dissolving 15–20 mg of the product in ≈0.8 mL of deuterated dimethylformamide (DMF‐d_7_), deuterated dimethyl sulfoxide (DMSO‐d_6_) or deuterated acetone (acetone‐d_6_).

^1^H Diffusion Ordered NMR Spectroscopy (DOSY) experiments were performed on a JEOL CZR 500 MHz spectrometer. The experimental observable is the attenuation of the echo, defined as the ratio of the NMR signal intensity *I(b,t)* after application of the pulse gradient to the intensity in the absence of the gradient *I*_0_. The decrease in intensity depends on the diffusion coefficient *D* according to Equation (2):

(2) $$\;\frac{I\left(b,t\right)}{I_{0}}=e^{\left(-Db^{2}t\right)}\;$$

where *b* is a variable dependent on the gradient pulse intensity (*g*) and its duration (*δ*) and *t* is the time interval during which molecules diffuse.^[^ ^60^, ^61^ ^]^ Through an exponential fitting of *I(b,t)* as a function of *g^2^t*, it is possible to calculate the value of *D*.

The number and weight average molecular weights (*M*_n_, *M*_w_) were determined by gel permeation chromatography (GPC), using a Jasco PU‐2089Plus liquid chromatograph equipped with two PL gel 5 µm mixed‐D columns, a Jasco RI‐2031 Plus refractive index detector and a Jasco UV‐2077Plus UV/vis detector. Measurements were carried out by using DMF as the mobile phase, at a flux of 1 mL min^−1^ and a temperature of 30 °C maintained by a Jasco CO 2063 Plus column thermostat. Polystyrene standards were used for calibration (400–400 000 g mol^−1^). The samples were filtered through a 0.2 µm PTFE filter.

Thermogravimetric analysis was performed by using a thermogravimetric analyzer Mettler TGA Q500. Samples of ≈5 mg in weight were prepared and analyzed under nitrogen inert flux of 80 mL min^−1^ by heating from 30 to 700 °C at 10 °C min^−1^.

Differential scanning calorimetry (DSC) analysis was performed with a TA Instruments Discovery DSC 250 calorimeter (Columbus, OH, USA). Samples weighing between 5 and 10 mg were dried under vacuum at room temperature and stored in a dry environment. Polymers were studied between −90 and 200 °C, at 10 °C min^−1^ in heating and cooling cycles. Glass transition temperatures (*T*_g_) with their specific heat changes (Δ*C*_p_) were taken at the inflection point of the devitrification step in the second heating scan. Melting temperatures (*T*_m_) were taken at the maximum of the corresponding endothermic peak and fusion enthalpy (Δ*H*_m_) as its integral in the curve of the second heating step.

The mechanical properties of the membranes were evaluated by stress‐strain tests. Dog bone‐shaped specimens (21.1 mm × 4.75 mm) were cut and conditioned for 24 h in deionized water at room temperature. The specimens were swabbed with filter paper. Stress‐strain curves were recorded at a grip separation rate of 2.5 mm min^−1^ with an INSTRON 5564 (Instron Corporation, Norwood, MA, USA).

Water uptake (*WU*) was determined according to the following procedure. The dry weight (*W*_dry_) of a piece of membrane was measured after being dried overnight under a vacuum at 60 °C. The membrane was then conditioned in deionized water for 24 h at room temperature and the wet weight (*W*_wet_) was measured after being wiped with filter paper. The water uptake was calculated according to Equation (3):

(3) $$\mathrm{WU}\left(\%\right)=\frac{W_{\mathrm{wet}}\;-\;W_{\mathrm{dry}}}{W_{\mathrm{dry}}}100$$

The ion exchange capacity (*IEC*_exp_) of a membrane was evaluated by using acid‐base titration.^[^ ^62^ ^]^ Specifically, the membrane was previously cut into pieces and dried under vacuum at 60 °C. The dried strips were weighed in a closed vessel and then immersed in 5 m NaCl and left overnight at room temperature to exchange H^+^ with Na^+^ ions. Afterward, the H^+^ concentration in the solution was titrated with 0.01 m NaOH. The endpoint was detected using the pHmeter method. *IEC*_exp_ was calculated according to Equation (4):

(4) $$\;IEC_{exp}=\frac{M_{NaOH}\;\times \;V_{NaOH\;}}{W_{dry}}$$

where *M*_NaOH_ and *V*_NaOH_ represent the molar concentration and the volume of NaOH used in the titration to reach the equivalent point, respectively.

### AQDS and Bromine Species Crossover

The AQDS/bromine crossover through the membrane was determined by electrochemical measurements. A glass H‐cell was assembled by placing the membrane between two glass half‐cells, secured with a clamp. One half‐cell contained a solution of 1.5 m H_2_SO_4_, a magnetic stir bar, a glassy carbon working electrode (WE), and an Ag/AgCl reference electrode (RE). The other half‐cell was filled with a solution of 1.5 m H_2_SO_4_ and a pre‐determined concentration of the analyte (0.5 m for AQDS and 1.5 m for HBr) with a platinum wire counter electrode (CE). The crossover rate of the analytes (mol s^−1^) was measured through potentiostatic measurements using a multi‐channel potentiostat/galvanostat (VMP3 from Bio‐Logic SA, Seyssinet‐Pariset, France). The working electrode potential was fixed at 1 V versus Ag/AgCl for HBr and −0.1 V versus Ag/AgCl for AQDS. The electric current between the WE and CE was recorded over time. The electric current between the WE and CE was recorded over time. The WE, acting as a sensor, was maintained at a specific potential to selectively oxidize or reduce the analyte. The current measured is directly proportional to the crossover rate, reflecting the number of analyte molecules oxidized or reduced on the WE surface. The crossover of AQDS and HBr through the membranes was normalized by the area and thickness of the membrane, and it was calculated according to Equation (5):

(5) $$\begin{array}{ccc} \mathrm{Cros}\mathrm{sover} & = & \frac{\mathrm{Cros}\mathrm{sover}\;\mathrm{rate}\;\left(\frac{\mathrm{mol}}{s}\right)}{A(cm^{2})}\;.\;d\left(\mathrm{cm}\right)\;\mathrm{with}\;\mathrm{Cros}\mathrm{sover}\;\mathrm{rate}\\ & = & \frac{\mathrm{Curr}\mathrm{ent}\;(A)}{96485\;\left(\frac{A\cdot s}{\mathrm{mol}}\right)\cdot z} \end{array}$$

where *z* represents the electrons exchanged during the oxidation and reduction processes equal to 2 for AQDS and 1 for HBr, *A* and *d* are the area and thickness of the membrane, respectively.

### Single RFB Cell Configuration

Membrane performance was determined by using a 4 cm^2^ active area single cell RFB. The cell is composed by two bipolar plates with interdigit flow field made of graphite (SGL carbon), which is highly chemically resistant, two copper made plates as current collectors, two end plates made of anodized aluminum with reactant input and output ports, and two polypropylene (PP) made plates as isolators. The single cell was assembled by sandwiching a membrane between two carbon cloth electrodes (Zolteck Tm Px35 satin weave 08). In order to have a good sealing and to balance the pressure distribution, Viton sheets (thickness 1 mm) were placed between the bipolar plates and the membrane. The RFB single cell was sealed up with a torque of 6.5 Nm.

### AQDS/Bromine Single‐Cell Battery

Potenstiostatic electrochemical impedance spectroscopy (PEIS) and galvanostatic charge and discharge experiments were conducted with a multi‐channel potentiostat/galvanostat. Charge and discharge cycling study was used to evaluate the performance of the battery at different current density values, whereby ten cycles were performed at 50 mA cm^−2^ and other ten cycles at 100. The experiment was carried out in a single‐cell test (4 cm^2^).^[^ ^51^ ^]^ The energy efficiency of the electrochemical cell was determined for cycles performed at the same current (galvanostatic). The electrolyte solutions were 0.5 m AQDS and 1.5 m HBr and the complexing agent solution was 0.165 m MEP. The electrolyte solution was cyclically pumped into the corresponding half‐cell by a volumetric pump (Masterflex, WVR International, Milan, Italy) and a constant flow rate of 150 mL min^−1^ was maintained.

### Synthesis of Graft Copolymers

In a typical procedure, pVDF (1.0 g), styrene (3.24 mL), CuCl (0.15 g), PMDETA (1.39 mL), and NMP (6.43 mL) were loaded into a 25 mL Carius tube equipped with a magnetic stirrer; the mixture was degassed by five freeze‐pump‐thaw cycles. The polymerization reaction was then carried out at 120 °C under vacuum. After 40 h, the reaction was stopped, and the crude product was precipitated three times from DMF solutions into methanol. The final graft copolymer, containing 30 mol.% Sty, was named pVDF‐*g*‐Sty30 and was characterized by ^1^H‐NMR and ^19^F‐NMR.

^1^H‐NMR (d_7_‐DMF, δ in ppm): 7.4−6.3 (aromatic protons of Sty), 3.3−2.8 (CH_2_CF_2_CH_2_CF_2_), 2.5−2.35 (CF_2_CH_2_CH_2_CF_2_), 2.3−1.2 (aliphatic protons of Sty).

^19^F‐NMR (d_7_‐DMF, δ in ppm): −91.0 to −93.0 (CH_2_CF_2_CH_2_CF_2_) (head‐to‐tail sequences), −94.6 (CH_2_CF_2_CF_2_CH_2_CH_2_CF_2_) (tail‐to‐tail sequences), −113.7 (CH_2_CF_2_CF_2_CH_2_) (head‐to‐head sequences), −116 (CH_2_CF_2_CF_2_CH_2_) (head‐to‐head sequences).

### Sulfonation Reaction

In a typical procedure, pVDF‐*g*‐Sty39 (3.1 g) and 96 wt.% H_2_SO_4_ (200 mL) were loaded in a three‐necked round bottom flask equipped with a magnetic stirrer and a bubble cooler. The reaction was carried out at 70 °C. After 4 h the reaction was stopped and the mixture was diluted with deionized water (DI). The functionalized polymer was filtered and then rinsed with DI; the operation was repeated 4 times until the rinsing waters were neutral. The purified graft copolymer, containing 23 mol.% Sty and 14 mol.% SSty, was named pVDF‐*g*‐(Sty23‐*co*‐SSty14) and was characterized by ^1^H‐NMR and ^19^F‐NMR (Figures 3 and 4).

^1^H‐NMR (d_6_‐DMSO, δ in ppm): 7.4−7.2 (aromatic *ortho* protons of SSty), 7.2−6.0 (*meta* and *para* protons of SSty and Sty), 3.2‐2.6 (CH_2_CF_2_CH_2_CF_2_), 2.3−2.0 (CF_2_CH_2_CH_2_CF_2_), 2.0−0.7 (aliphatic protons).

^19^F‐NMR (d_6_‐DMSO, δ in ppm): −91.0 to −93.0 (CH_2_CF_2_CH_2_CF_2_) (head‐to‐tail sequences), −95.7 (CH_2_CF_2_CF_2_CH_2_CH_2_CF_2_) (tail‐to‐tail sequences), −114.6 (CH_2_CF_2_CF_2_CH_2_) (head‐to‐head sequences), −117.0 (CH_2_CF_2_CF_2_CH_2_) (head‐to‐head sequences).

### Preparation of Proton Exchange Membranes

In a typical procedure, a sulfonated graft copolymer (785 mg) was dissolved in DMF (6.3 mL) at room temperature under magnetic stirring. The solution was then cast by using an automatic film applicator (Automatic Film Applicator TQC Sheen AB4120, TQC Sheen Italy, Seregno, Italy), by setting the thickness of the ruler at 700 µm and using a speed of 5 mm s^−1^. Afterward, the membrane was heated at 50 °C for 5 h to complete solvent evaporation. The final thickness of the dry membrane was 70–80 µm.

## Conflict of Interest

The authors declare no conflict of interest.

## Supporting information



Supporting Information

## Data Availability

The data that support the findings of this study are available from the corresponding author upon reasonable request.
