# Supplementary material for: Sulfonated Styrene‐Grafted Polyvinylidene Fluoride Copolymers for Proton Exchange Membranes for AQDS/Bromine Redox Flow Batteries
Source: Macromol Rapid Commun. 2024 Dec 27;46(5):2400852. doi: 10.1002/marc.202400852 (PMC11884226; doi:10.1002/marc.202400852)
Supplement: Supplementary file 1 — Supporting Information [file MARC-46-2400852-s001.docx]

# SUPPORTING INFORMATION

**Francesca Niccolai**^1,2^**, Elisa Guazzelli**^1^**, Andrea Cesari,**^1^ **Zakaria El Koura^2^, Ilaria Pucher**^2^**, Giancarlo Galli***^1^**, Elisa Martinelli***^1^

^1^Department of Chemistry and Industrial Chemistry, University of Pisa, 56124, Pisa, Italy

E-mail: giancarlo.galli@unipi.it, elisa.martinelli@unipi.it

^2^Green Energy Storage (GES), 38123 Povo, Trento, Italy

**Figure S1**. ^1^H-NMR spectra of pSty (1), pVDF-*g*-Sty51 (2), pVDF-*g*-Sty49 (3), pVDF-*g*-Sty39 (4), pVDF-*g*-Sty29 (5), pVDF (6). d = dimethylformamide, a = acetone, w = water

**

**Figure S2**. ^19^F-NMR spectra of pVDF-*g*-Sty51 (1), pVDF-*g*-Sty49 (2), pVDF-*g*-Sty39 (3), pVDF-*g*-Sty29 (4), pVDF (5).

**Table S1.** Diffusion coefficient values (*D*, m^2^s^-1^) for different polymers: pVDF, pVDF-*g*-Sty39 and pVDF-*g*-Sty49 (500 MHz, DMF-d_7_, 25 °C, 15 mg/mL).

| Polymer | D  (m^2^s^-1^) |
| --- | --- |
| pSty (19000 g/mol) | (7.6±0.3)⋅ 10^–10^ |
| pSty(233000 g/mol) | (1.2±0.1)⋅10^–10^ |
| pVDF | (1.8±0.3)⋅10^–11^ |
| pVDF-g-Sty39 | (6.4±0.2)⋅10^–11^ |
| pVDF-g-Sty49 | (2.8±0.1)⋅10^–11^ |

**Figure S3.** GPC curves of pVDF-*g*-Styx in DMF obtained by RI detection.


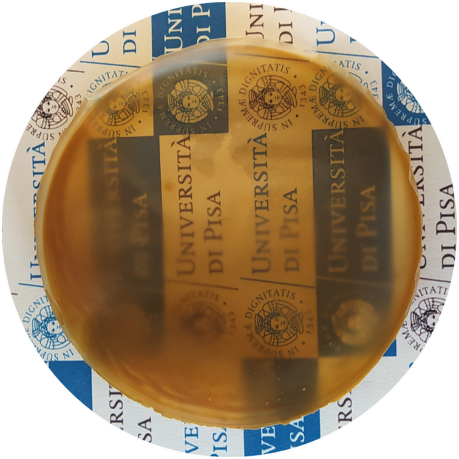


**Figure S4.** Image of a cast film of a sulfonated styrene graft copolymer.

**Table S2.** Thermogravimetric data of the pVDF-*g*-Styx grafted copolymers.

| Graft copolymer | T^a^_onset1_  (°C) | T^b^_max1_  (°C) | T^c^_max2_  (°C) | Residue^d^ (wt%) |  |
| --- | --- | --- | --- | --- | --- |
| pVDF |  |  | 478 | 34 |  |
| pVDF-g-Sty29 | 406 | 426 | 480 | 15 |  |
| pVDF-g-Sty39 | 399 | 422 | 465 | 16 |  |
| pVDF-g-Sty49 | | 407 | 431 | 475 | 13 |
| pVDF-g-Sty51 | | 403 | 425 | 473 | 17 |
| pSty | | 364 | 397 |  | 3 |

^a)^ Initial thermal degradation temperature. ^b)^ Temperature of the maximum weight loss rate associated with the first degradation step. ^c)^ Temperature of the maximum weight loss rate associated with the second degradation step. ^d)^ Residue at 700°C.

**Table S3.** Thermogravimetric data of sulfonated styrene graft copolymers.

| Polymer | DS  (%) | T_onset_^a^  (°C) | T_max_^b^  (°C) | T_max_^c^  (°C) | Residue^d^  (wt%) |
| --- | --- | --- | --- | --- | --- |
| pVDF-g-(Sty6-co-SSty14) | 70 | 358 | 408 | 449 | 27 |
| pVDF-g-(Sty14-co-SSty16) | 53 | 398 | 420 | 460 | 28 |
| pVDF-g-(Sty23-co-SSty14) | 38 | 395 | 413 | 441 | 25 |
| pVDF-g-(Sty26-co-SSty14) | 35 | 373 | 408 | 453 | 21 |

^a)^ Initial thermal degradation temperature. ^b)^ Temperature of the maximum weight loss rate of the first region. ^c)^ Temperature of the maximum weight loss rate of the second region. ^d)^ Residue at 700 °C.


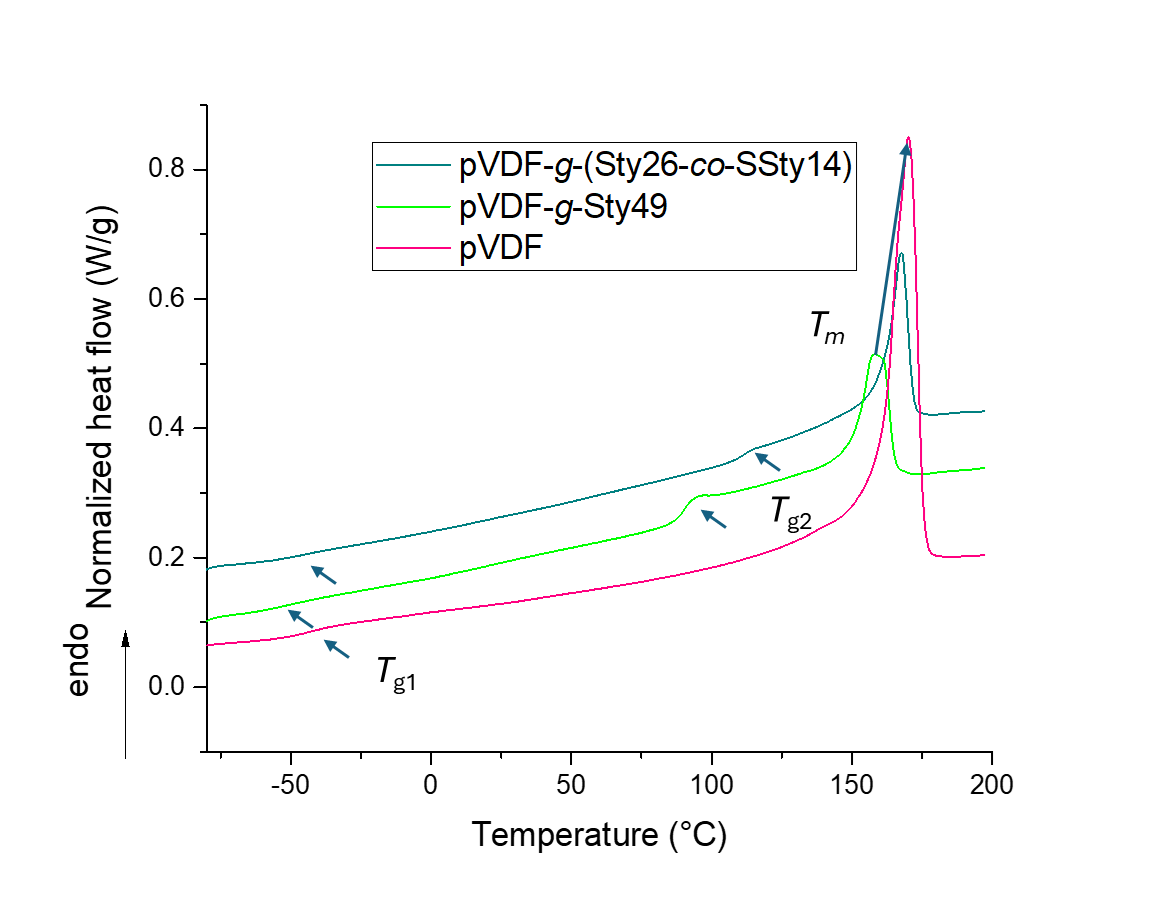


**Figure S5.** DSC second heating curves for pVDF, pVDF-*g*-Sty49 and pVDF-*g*-(Sty26-*co*-SSty14).

**Table S4.** Mechanical properties of sulfonated styrene graft copolymers membranes.

| Polymer | DS | E ^a^  (MPa) | σ_max_  (MPa) | ε  (%) |  |
| --- | --- | --- | --- | --- | --- |
| pVDF-g-(Sty6-co-SSty14) | 70 | 1085±292 | 23±9 | 7±5 |  |
| pVDF-g-(Sty14-co-SSty16) | 53 | 739±203 | 18±6 | 6±1 |  |
| pVDF-g-(Sty23-co-SSty14) | 38 | 678±128 | 19±3 | 3±1 |  |
| pVDF-g-(Sty26-co-SSty14) | | 35 | 220±131 | 7±4 | 7±5 |
| pVDF | |  | 1142 ±285 | 27± 8 | 198 ± 52 |

^a)^ Elastic modulus determined as the slope of the linear range of the stress-strain curve.
